# Metagenomics: The Next Culture-Independent Game Changer

**DOI:** 10.3389/fmicb.2017.01069

**Published:** 2017-07-04

**Authors:** Jessica D. Forbes, Natalie C. Knox, Jennifer Ronholm, Franco Pagotto, Aleisha Reimer

**Affiliations:** ^1^National Microbiology Laboratory, Public Health Agency of Canada, WinnipegMB, Canada; ^2^Department of Medical Microbiology and Infectious Diseases, University of Manitoba, WinnipegMB, Canada; ^3^Department of Food Science and Agricultural Chemistry, Faculty of Agricultural and Environmental Sciences, McGill University, MontrealQC, Canada; ^4^Department of Animal Science, Faculty of Agricultural and Environmental Sciences, McGill University, MontrealQC, Canada; ^5^Bureau of Microbial Hazards, Food Directorate, Health Canada, OttawaON, Canada; ^6^Listeriosis Reference Centre, Bureau of Microbial Hazards, Food Directorate, Health Canada, OttawaON, Canada

**Keywords:** metagenomics, targeted-amplicon, food safety, public health, culture-independent diagnostic test, next-generation sequencing, antimicrobial resistance, molecular epidemiology

## Abstract

A trend towards the abandonment of obtaining pure culture isolates in frontline laboratories is at a crossroads with the ability of public health agencies to perform their basic mandate of foodborne disease surveillance and response. The implementation of culture-independent diagnostic tests (CIDTs) including nucleic acid and antigen-based assays for acute gastroenteritis is leaving public health agencies without laboratory evidence to link clinical cases to each other and to food or environmental substances. This limits the efficacy of public health epidemiology and surveillance as well as outbreak detection and investigation. Foodborne outbreaks have the potential to remain undetected or have insufficient evidence to support source attribution and may inadvertently increase the incidence of foodborne diseases. Next-generation sequencing of pure culture isolates in clinical microbiology laboratories has the potential to revolutionize the fields of food safety and public health. Metagenomics and other ‘omics’ disciplines could provide the solution to a cultureless future in clinical microbiology, food safety and public health. Data mining of information obtained from metagenomics assays can be particularly useful for the identification of clinical causative agents or foodborne contamination, detection of AMR and/or virulence factors, in addition to providing high-resolution subtyping data. Thus, metagenomics assays may provide a universal test for clinical diagnostics, foodborne pathogen detection, subtyping and investigation. This information has the potential to reform the field of enteric disease diagnostics and surveillance and also infectious diseases as a whole. The aim of this review will be to present the current state of CIDTs in diagnostic and public health laboratories as they relate to foodborne illness and food safety. Moreover, we will also discuss the diagnostic and subtyping utility and concomitant bias limitations of metagenomics and comparable detection techniques in clinical microbiology, food and public health laboratories. Early advances in the discipline of metagenomics, however, have indicated noteworthy challenges. Through forthcoming improvements in sequencing technology and analytical pipelines among others, we anticipate that within the next decade, detection and characterization of pathogens via metagenomics-based workflows will be implemented in routine usage in diagnostic and public health laboratories.

## Introduction

The incidence and impact of foodborne illness constitutes a significant global issue to public health. Foodborne illness affects one in eight Canadians annually, resulting in an estimated 4 million infections, 11,600 hospitalizations and 238 deaths (Thomas et al., 2015); the leading causes of known foodborne infections include norovirus (65%), *Clostridium perfringens* (11%), *Campylobacter* spp. (8%), and non-typhoidal *Salmonella* spp. (5%). According to Health Canada, approximately 2.4 million cases or 60% of foodborne illness are attributed to unknown causes versus only 1.6 million cases or 40% causatively linked to 30 recognized foodborne microbes which include bacteria, viruses and parasites (Thomas et al., 2015). The detection of foodborne enteric pathogens and hence diagnosis of foodborne disease, historically (and still considered the gold standard) have been conducted via culture-dependent techniques, that is, the physical isolation of a bacterial pathogen. Though recognized for some time, the utilization of CIDTs has been increasing throughout the last decade, effectively transforming clinical and food microbiology laboratories (Jandaa and Abbottb, 2014).

In clinical diagnostic laboratories, pathogen detection is increasingly contingent upon the analytic application of CIDTs, which include nucleic acid (e.g., PCR) and antigen-based tests (e.g., ELISA) among others. The extensive adaptation of CIDTs to clinical and food settings is largely reliant on inherent advantages over traditional culture-dependent diagnostic tests; use of CIDTs offers a considerably faster TAT which is crucial for (i) clinical decision-making and decreasing the unnecessary use of broad-spectrum or ineffective antimicrobials, (ii) early outbreak detection and control, and (iii) food industry release or recall of products. Moreover, most conventional CIDTs require less technical expertise and in the long-term may offer a more cost effective alternative.

The clinical use of CIDTs presents the potential to improve disease detection. First, CIDTs are reportedly more sensitive and specific than culture (Hanson and Couturier, 2016; Steyer et al., 2016). Second, reduced complexity of CIDTs allows for rapid testing thereby allowing for a higher throughput of biological specimens to be tested. Third, CIDTs can identify non-culturable or fastidious microbes such as *Campylobacter* spp. (Fitzgerald et al., 2016) or noroviruses (Jones et al., 2014). Lastly, CIDTs enhance the ability to identify polymicrobial or complex infections. Not only are CIDTs attractively useful in the clinical microbiology laboratory but also in recent years their utilization has become widespread for routine and rapid detection of common pathogens in other sectors including public health and food safety laboratories in addition to *in situ* testing of food processing establishments and agricultural sites. Traditional diagnostics, however, rely upon tests that are tailored to the etiological agent associated with a particular syndrome.

Next-generation sequencing technology has effectively transformed infectious disease research throughout the last decade. High-throughput laboratory techniques are bypassing onerous testing via complement or replacement of conventional microbiological, molecular and serological tests for identifying, typing and characterizing pathogens. WGS of cultured isolates has been extensively employed for pathogen characterization, outbreak detection, phylogenomics and microbial genome wide association studies and thus has progressed from the proof-of-principle phase to implementation in routine foodborne surveillance and outbreak response (Jackson et al., 2016).

**FIGURE 1:**
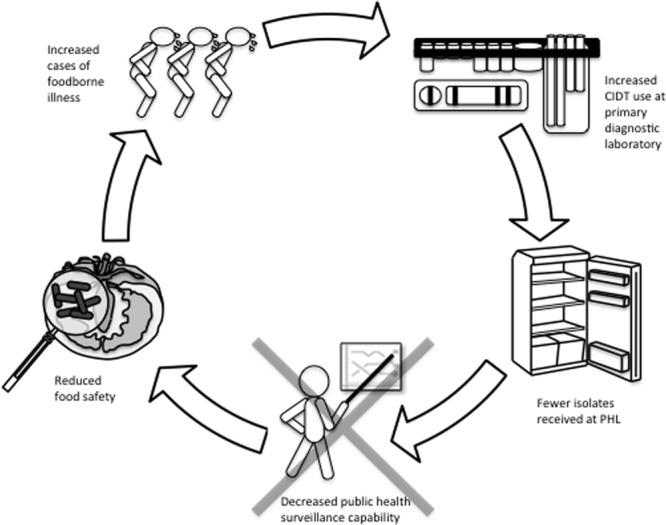
Cycle of how CIDT upsurge in clinical laboratories may have compound effects on the food industry. Increased usage of culture-independent syndromic panels to diagnose cases of acute gastroenteritis is expected to result in fewer microbial cultures referred to public health laboratories. Fewer isolates at public health laboratories will limit surveillance abilities with negative impacts to (i) monitoring trends in AMR, (ii) detection and response to food safety incidents and outbreaks, and (iii) source attribution. Consequently, the food supply in Canada and potentially other countries, may be exposed to an increase in foodborne illness. In turn, the repercussions of this cycle in the absence of reflex cultures could have serious implications at all levels of foodborne disease management.

Current public health infectious disease surveillance methodologies are generally reliant upon the frontline laboratory to refer pure culture isolates to their local or provincial public health laboratory. As we progressively enter a culture-independent era for infectious disease diagnostics, public health laboratories will inevitably receive fewer isolates. Hence, performing appropriate subtyping and AST assays to identify and track foodborne outbreaks will be difficult. Inadequate surveillance measures have the potential to negatively affect food safety via the inability to identify outbreaks and perform source attribution studies of contaminated foods. We expect this will result in an increase of contaminated foods lingering on the market thereby causing more cases of undetected foodborne outbreaks (Carleton and Gerner-Smidt, 2016). **Figure 1** shows a schematic representation of the effect of CIDTs on the food industry.

Employment of molecular methods that are highly informative will improve the all-encompassing issues associated between primary diagnostic and public health laboratories. Metagenomics for example offers the advantage of a less biased pathogen detection methodology through direct sequencing of the specimen’s extracted DNA. This approach has the potential to capture a thorough representation of the microbial community (with some limitations; discussed below) thus eliminating the requirement for pure culture. Metagenomics and similar techniques traditionally have been applied to interrogate microbiomes of a particular ecological niche through sequencing of all nucleic acids recovered from a sample (Eckburg et al., 2005; Huttenhower et al., 2012; Mason et al., 2014). Moreover, a number of clinically relevant applications stand to benefit from such data – rapid identification of the etiological agent (known or novel) and gene content including virulence and AMR, or inferring functional pathways to elucidate multifaceted illnesses.

**FIGURE 2:**
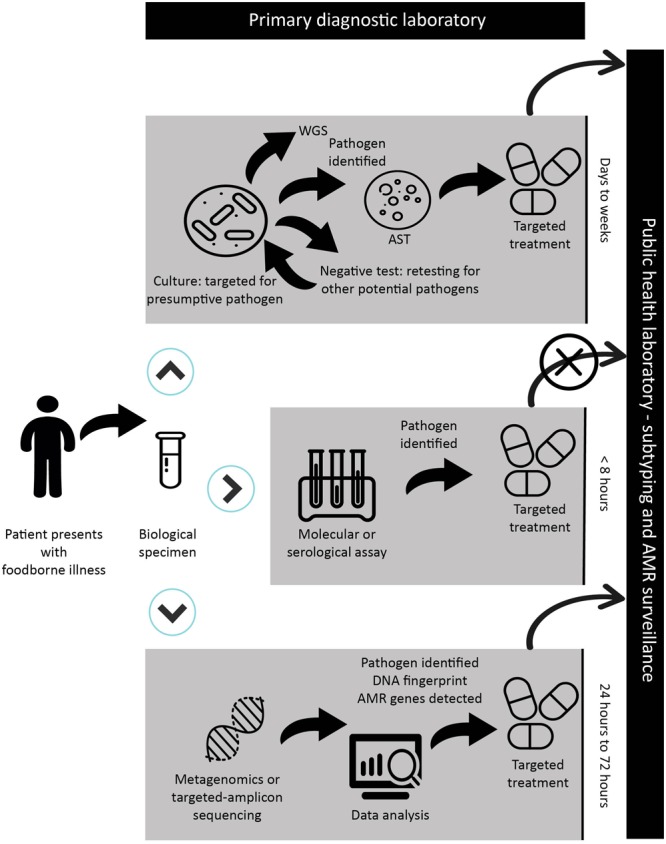
Schematic representation of a sample workflow with timelines from patient presentation at a primary health care facility through the diagnostic process and public health surveillance in the case of a reportable disease. Depending on the facility and its use of CIDT, the biological specimen will be submitted for culture-based assay and biochemical testing; CIDT testing including molecular or serological assays; or NGS-CIDTs when all other assays have failed. In the case of a CIDT workflow, a positive assay from the clinical specimen is discarded leading to the inability to forward an isolate or specimen for public health surveillance. The prospect of NGS-CIDT use in clinical laboratories has the advantage of replacing a variety of diagnostic tests. The application of NGS-CIDT may ultimately negate the need to retain clinical specimens and isolates for public health laboratory surveillance. In the context of NGS-CIDT, while we provide a general timeframe, it should be stressed that sequencing and analysis times are highly variable and dependent on the sequencing platform and bioinformatics analysis. Importantly, while this workflow is illustrated to reflect foodborne diseases, it is easily applied to other infectious diseases, though in the case of culture-based methods, timelines would be drastically different for some pathogens.

Laboratory methods that detect and identify pathogens serve two critical functions: clinical decision-making (individual level) and public health decision-making (population level). While traditional culture-based methods meet both of these needs simultaneously, CIDTs are exclusively designed to improve clinical decision making only. This leaves critical public health activities at great risk. A summary of techniques used in primary diagnostic labs is illustrated in **Figure 2**. Herein, we will review the current state of CIDTs as they relate to foodborne illness and food safety. We will furthermore discuss the diagnostic utility of metagenomics and comparable detection techniques in clinical microbiology, food and public health laboratories.

## Current State of Conventional Culture-Independent Diagnostic Tests in Clinical and Food Microbiology Laboratories

Traditional culture-based methods for detecting enteric pathogens in biological specimens, food processing environments or foods is dependent on the growth of viable and culturable microbes; biochemical tests and subtyping are further needed to confirm microbe identification and to establish genetic linkage, respectively. Though sensitive, relatively easy and inexpensive, culture-based methods are laborious and ineffective for non-culturable or fastidious microbes. The diagnostic potential of CIDTs to circumvent the demanding task of culturing and subtyping pathogens is a widely attractive alternative.

### Conventional Culture-Independent Diagnostic Tests

Though globally, data is limited regarding the current use of CIDTs in clinical and food laboratories, many clinical laboratories in the US are reportedly in the process of converting to the use of CIDTs for the identification of enteric microbes (Shea et al., 2017). According to the CDC, significant increases in the amount of enteric infections diagnosed in the US entirely by CIDTs were reported in 2015 compared to year’s prior (2012–2014). In particular, increases in positive CIDTs were reported for 4 enteric pathogens: *Campylobacter* (92%), *Shigella* (284%), *Salmonella* (247%), and STEC (120%; Huang et al., 2016). We expect the increased use of CIDTs is less dramatic in Canada, with suspected regional differences, due to health-care system differences, though there is a paucity of published data to support this theory. For example, the Canadian health care system is not driven by private insurance, as is the case in the US, but rather, is publicly funded. To establish trends of CIDT uptake in Canada, surveys will be required to evaluate their usage in primary diagnostic laboratories. We anticipate that the percentage of infections diagnosed with CIDTs will continue to rise in clinical and food microbiology laboratories in developed countries in the coming years.

This past decade has seen a drastic expansion in the diagnostic application of available and validated CIDTs (Doggett et al., 2016; Shea et al., 2017). There is a wide degree of variation amongst implementation of CIDTs in diagnostic laboratories ranging from singleplex PCR assays to complete laboratory automation; thus, microbiologists are able to yield clinically actionable results of superior quality ultimately benefiting patient health and outcome. Conventional CIDTs can be assigned to one of two methodologies: nucleic acid-based methods and antigen-based tests. The use of nucleic acid-based assays to identify enteric pathogens presents with several advantages over culture-dependent tests in clinical and food settings. Their use allows for high levels of sensitivity and specificity and offers an added benefit in their ability to detect toxin-producing genes or other important biomarkers. Numerous nucleic acid assays are in routine use including PCR (singleplex, multiplex, quantitative, quantitative reverse-transcription, real-time), amplification such as LAMP and NASBA, DNA microarray, microfluidic chip and MALDI-TOF mass spectrometry. Alternatively, antigen-based tests for enteric pathogen detection include ELISA and LFA; reviewed in Zhao et al. (2014). A detailed description of conventional CIDTs is outside the scope of this review and we refer readers to an exhaustive review of CIDTs (Doggett et al., 2016).

A growing number of US FDA – approved syndromic panels for multiple pathogen detection in addition to laboratory-developed tests have facilitated the upsurge of CIDT application for acute gastroenteritis in clinical laboratories. These panels employ PCR to detect unique DNA sequences to enable identification of enteric pathogens. Several commercially available gastrointestinal multiplex panels are in current use and include BDMax, FilmArray, Luminex, Prodesse, and Verigene. Gastrointestinal CIDT panels are advantageous over culture-based diagnostics for several reasons. First, the gastrointestinal panels are designed to detect multiple pathogens in a single assay, in some cases (e.g., FilmArray) up to 22 microbes. Commercially available assays differ in their microbial targets detecting distinct subsets of bacteria, viruses and/or parasites. Second, the various assays have different TAT – most ranging from 1 to 4 h – though even the most time-intensive assays (8 h) are considerably faster than stool culturing which can take 2–5 days dependent on the microbe being cultured. Third, multiplex panels retain the ability to detect polymicrobial infections, which is particularly relevant in clinical settings. Lastly, multiplex panels are highly sensitive (90–100%) and specific (98%) (Buchan et al., 2013; Navidad et al., 2013; Onori et al., 2014; Buss et al., 2015; Harrington et al., 2015).

## Next-Generation Sequencing As A Diagnostic Assay

### Whole Genome Sequencing

Next-generation sequencing and other high-throughput laboratory techniques are circumventing laborious testing by active replacement or complement to traditional microbiological and molecular tests for identifying, typing and characterizing pathogens. An increase in read-length, output and quality of short-read NGS technologies has made it possible to apply WGS as a genomic surveillance system of foodborne diseases and outbreak management (Köser et al., 2012). In most cases, WGS provides higher discriminatory power than the combined effort of multiple conventional typing assays such as PFGE, MLST, MLVA, phage typing, virulence typing, and AST. Moreover, WGS is more reliable in a shorter time period and can be performed in a single comprehensive procedure thus allowing for rapid and sensitive pathogen identification and similarly for reporting to the food industry and government responsible for public health decision-making (Ashton et al., 2016; Jackson et al., 2016).

For some clonal microbes, molecular typing methods have proven unable to accurately discriminate genetically distinct isolates. As an example, in past outbreak investigations, highly clonal *Salmonella enterica* serovars such as *S. enteritidis* have been challenging to resolve using PFGE (the gold standard typing method). Taylor et al. (2015) reviewed seven epidemiologically supported clusters and revealed that SNV analysis was able to accurately discriminate cluster isolates from sporadic and suspect samples with high epidemiological concordance. PFGE results would have suggested that distinct outbreak isolates with no epidemiological link might have originated from the same source. The low resolution of PFGE was also exemplified in the 2008 listeriosis outbreak whereby suspected cases emerged with a mixture of two distinct but closely related PFGE patterns (Gilmour et al., 2010); WGS analysis revealed that isolates from both PFGE patterns were genetically similar apart from a large prophage responsible for the 40 kb band shift difference seen in the PFGE patterns of suspected isolates. WGS was only a research tool in those early days of public health genomics; however, its use in this large-scale outbreak was able to confirm that outbreak strains subtyped by either PFGE pattern were genetically similar apart from the insertion of a large prophage. The inclusion or exclusion of clinical strains associated with the outbreak and the resulting case definition can alter the case epidemiology and food safety investigation efforts.

Several NGS sequencing platforms are available; each with their own advantages and disadvantages differing in sequencing time, read length, cost and others, reviewed elsewhere (Mardis, 2017). At present, WGS, though still dependent on the presence of isolates, represents one of the predominant investigative tools to rapidly and accurately identify microbes, perform subtyping, cluster epidemiological relevant isolates in outbreak investigations and detect AMR genes and virulence profiles. The use of WGS has also proven exceedingly useful in retrospective outbreak investigations. In 2013, PulseNet USA incorporated WGS as a surveillance tool for all *Listeria monocytogenes* isolates (Jackson et al., 2016) and is poised to begin WGS of *Campylobacter*, STEC and *Salmonella*; PulseNet Canada is similarly performing routine WGS of all *L. monocytogenes* and intends to begin sequencing all *Salmonella* isolates routinely in 2017. Moreover, PulseNet Canada also performs WGS on select cluster or outbreak investigations of STEC and other organisms.

### Diagnostic Metagenomics and Comparable Detection Techniques

Diagnostic approaches including culture and non-NGS CIDTs such as PCR or serology continue to represent the gold standard for infectious disease diagnostics. Each of these methods, however, is disadvantaged in that they represent a targeted detection methodology and hence *a priori* knowledge or hypotheses to identify the etiological agent in the sample are required. It has been suggested that the limited capacity of conventional diagnostics including culture and non-NGS CIDTs are partly responsible for failing to detect an etiological culprit in a considerable number of cases (Denno et al., 2012). High-throughput targeted-amplicon and shotgun metagenomics sequencing methods circumvent this limitation via broad-range detection of either a subset of microbes (targeted-amplicon) or all microbes (shotgun metagenomics). Further, diagnostic shotgun metagenomics offers an added advantage with the possibility to identify previously uncharacterized microbes or emergent and novel pathogens (Forster et al., 2016).

The dramatic increase of microbiome research in the last decade is effectively driven by the widespread usage of high-throughput DNA sequencing and the associated decrease in sequencing cost. In the context of the microbiome, NGS allows for the complete description of all genomic content of microbial communities (e.g., bacterial, viral, eukaryotic microbes) in a technique referred to as shotgun metagenomics (Sharpton, 2014). Though most microbiome studies are fundamentally designed to describe commensal populations (The NIH HMP Working Group, 2009) or alternatively, to investigate dysbiosis in distinct human body compartments affected by disease (Forbes et al., 2016), or to determine the efficacy of prebiotics (Alfa et al., 2017) and others, metagenomics can similarly be utilized for the identification of pathogens in clinical (Mongkolrattanothai et al., 2017) or food (Aw et al., 2016) samples. Like other molecular or serological CIDTs, the detection of microbes in metagenomics is independent of culture (Schloss and Handelsman, 2005) in contrast to WGS, which is also reliant on a pure culture (Hasman et al., 2014). Metagenomics and comparable detection techniques generate immense quantities of large-scale sequence data thus bioinformatics and/or computational approaches are required to assign sequences to particular microbes, microbial functions or other descriptors of relevance (Sharpton, 2014).

The majority of microbiome studies to date have focused specifically on the bacterial portion of microbiomes rather than characterizing the microbial communities across all domains of life in addition to viruses (shotgun metagenomics). As such, these analyses are often performed by high-throughput targeted-amplicon sequencing of a universal phylogenetically informative genetic marker; the 16S rRNA gene is most commonly used though other markers are similarly able to discriminate between prokaryotes including *cpn60* (Schellenberg et al., 2016), *rpoB* (Case et al., 2007), 23S rRNA (Anthony et al., 2000), and others. Universal targets for eukaryotic organisms include the 18S rRNA gene (Kataoka et al., 2016) or alternatively, for specifically characterizing fungal populations the ITS is frequently used (Argenio et al., 2016).

While the science community has persistently been using the term metagenomics interchangeably to describe both high-throughput targeted-amplicon and shotgun metagenomics studies to profile microbial populations, it is important to make a clear distinction between both methodologies. Targeted-amplicon and shotgun metagenomics sequencing each present with advantages and disadvantages (**Table 1**). Features differentiating the two methods include targeted microbial community, associated costs and computational and technical expertise requirements among others. Thus, the decision to use one approach versus the other should be made with careful consideration and will be highly dependent on the research and/or diagnostic goals and hypotheses in question (**Table 2**). In this regard, targeted-amplicon assays are more apt for describing a specific group of microbes (e.g., bacteria) whereas a shotgun metagenomics approach is more suitable for characterizing the entirety of microbial DNA in a given sample limited by sequencing technology used and sample matrix with high host DNA-containing material.

**Table 1:** Summary of the advantages and disadvantages to each high-throughput sequencing approach for unbiased detection.

	Targeted-amplicon sequencing		Shotgun metagenomics sequencing	
	Advantages	Disadvantages	Advantages	Disadvantages
Microbial target(s) of interest	• Target is specific to a particular microbial group (e.g., 16S rRNA common for bacteria, archaea, 18S rRNA for eukaryotes, ITS for fungi, RdRP for RNA viruses).	Requires *a priori* knowledge for microbial group target.	•Can sequence all DNA in a given sample (e.g., bacteria, archaea, eukaryotes, parasites, and viruses).	Virome assays require complex sample and nucleic acid work-ups.
Abundance profiling	Can use relative abundance changes to compare microbiomes across different samples or treatments.	Universal target chosen may be present in varying copy numbers across different taxa (e.g., 16S rRNA). PCR amplification bias, primer bias and errors.	Universal markers can be inferred from metagenomics datasets.	• High abundance of host DNA can make it challenging to sequence low abundance microbial DNA.
	• Can capture abundance of rare taxa provided that sequencing depth is sufficient.	• Absolute abundance difficult to impute.		• Low abundance taxa difficult to identify. Can be difficult to accurately bin each sequence to a genome.
Taxonomic assignment	• Relatively easy to taxonomically classify sequences using a variety of validated tools and curated databases.	• Databases can be self-limiting and have the potential to exclude novel microbes.	• Plethora of software using phylogenetically informative gene markers.	• High proportion of taxonomically uninformative sequences are discarded.
		• Universal targets within microbial groups can give variable taxonomic classifications.		• Availability and access to comprehensive and curated databases across all microbial groups limited.
		• Taxonomic resolution variable – species level identification should be interpreted with caution.		
Cost	•Low cost		• Can be carried out on most bench-top sequencers and sequencing platforms.	• Can be cost prohibitive depending on the sequencing depth, sample type, and microbe(s) of interest.
	• Can be carried out on most bench-top sequencers and sequencing platforms.			• If high host DNA is expected or interest is in the low-abundance microbes or rare taxa, use of a higher throughput sequencer (Illumina HiSeq), may be required.
Computational requirements	• Most analysis steps can be carried out on a modern desktop.	• Large datasets (high sample number and/or sequencing coverage) may require access to a high performance computing cluster dependent on analytical pipeline chosen.	• Cloud computing services are available for metagenomics data analysis for those without access to a high performance computing cluster.	High performance computing environment absolutely necessary.
				• Cloud computing – potentially cost-prohibitive and might not have all available pipelines and/or software.
				• Data privacy and sensitivity may prohibit the use of commercial cloud computing services.
Technical expertise	• Moderate to high technical expertise is required depending on the analytical pipeline chosen.			• High technical expertise required.

**Table 2:** Overview of appropriate usage for each unbiased high-throughput sequencing approach.

Study goals/purpose	Suggested sequencing approach
Characterization of a particular microbial group (excluding viruses) in sample(s)	High-throughput targeted-amplicon sequencing; utilize shotgun metagenomics sequencing if interested in high taxonomic resolution above genus level.
Characterization of all microbial DNA in sample(s)	Metagenomics shotgun sequencing.
Pathogen detection	Dependent on the sample: • If the etiological agent is suspected to be of viral origin a shotgun metagenomics approach is warranted. • If the sample type contains a high host DNA load (e.g., blood) should consider a targeted-amplicon or deep shotgun metagenomics sequencing approach. The latter may be cost prohibitive and require access to a high-throughput sequencer (e.g., Illumina HiSeq). • Low biomass samples (e.g., BAL/CSF), might require a targeted-amplicon sequencing approach initially. Shotgun metagenomics sequencing may not be able to sequence the infectious agent adequately (e.g., only a few sequences produced which may only yield a confounding signal).
Functional profiling	Functional profiles can be inferred with a targeted-amplicon sequencing approach, however, results should be interpreted with caution due to the limitations of inferring gene function with universal targets. A shotgun metagenomics approach would yield more appropriate and reliable conclusions.
SNV or clonal isolate detection studies	Shotgun metagenomics sequencing.
Novel microbial identification and characterization	Targeted-amplicon sequencing relies on curated databases of known microbes and may not be able to adequately analyze novel microbes in an unbiased technique. Shotgun metagenomics would be recommended.

*BAL, bronchoalveolar lavage; CSF, cerebrospinal fluid; SNV, single nucleotide variant.*

### Metagenomics and Comparable Detection Techniques in Food Safety

**FIGURE 3:**
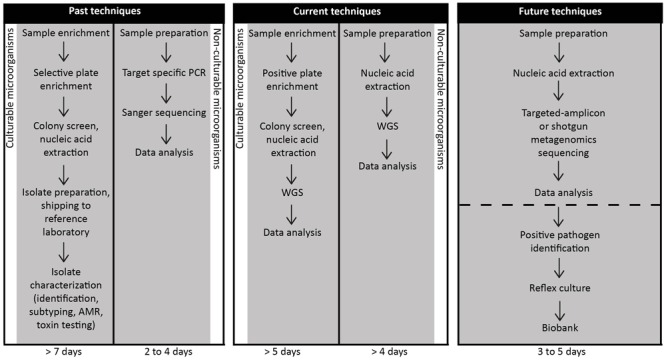
Timelines for the detection of foodborne pathogens using past, current or future techniques implemented in food microbiology laboratories. Foodborne pathogen detection historically and at present requires prior suspicion of the suspected etiological agent. Food samples using either past or current techniques are worked up via one of two methods dependent on the culturability of the presumptive pathogen. Time to results is variable with timeframes ranging from two to more than 7 days. We propose that metagenomics and targeted-amplicon assays will be executed in food microbiology laboratories in the near future. For non-culturable pathogens such as norovirus, metagenomics approaches have already been developed to recover the pathogens (Nasheri et al., 2017) in as few as 3 days. As metagenomics and comparable detection techniques are adapted for routine use in food microbiology laboratories for both culturable and non-culturable pathogens, this data will shorten the time to foodborne pathogen identification and secondly, to issue a recall if necessary. As these methods become more common in food microbiology laboratories, sample preparation technologies will change to help shorten and possibly eliminate the need for enrichment to increase the desired target. It will be important, however, to simultaneously perform reflex culturing of the pathogen for archiving and potential litigations associated with the investigation.

Culture-based techniques are still considered the gold standard in the food industry, including sectors such as business operators, government regulatory agencies and national or international compliance testing. Countries such as Canada have policies in place that require quantitative (Pagotto et al., 2011a) or qualitative (Pagotto et al., 2011b) approaches to determine viable pathogens such as *L. monocytogenes*^1^. This is attributed to how foods are categorized based on the ability to support (or not), the priority assigned to high-risk foods and the ability of the pathogen to survive during the shelf life of the food^1^. Moreover, the target pathogen is often in such low numbers that in the presence of the background microbiota, enrichment is required – and culturing is the most effective strategy (Gill, 2017). While a molecular fingerprint *per se* is not currently required for compliance activity, it is invaluable to epidemiological investigations and for source attribution. As such, current approaches necessitate a physical isolate in order to generate molecular fingerprints. Further, it is important to note that more than one “molecular type” may be implicated in an outbreak and so, culture-dependent technologies continue to have an important role. This was shown in the *Listeria* cantaloupe outbreak where multiple serovars and five different molecular subtypes were found (McCollum et al., 2013). It is anticipated that bioinformatics will help play a role in being able to differentiate the different molecular types present in a single food item (unlike a clinical specimen that tends to be contaminated with a single type). Molecular techniques that are currently used to compare isolates during an outbreak investigation, such as PFGE, ribotyping and gene-specific PCR, require an isolate as a starting point (Ronholm et al., 2016); these procedures would generally take approximately 7 days (**Figure 3**). If however, a high-sensitivity metagenomics methodology, capable of reliably detecting foodborne pathogens in samples with high-levels of background microbiota were developed, the time for outbreak recognition, causative agent sub-typing, secondary analyses for virulence and AMR genes and source attribution would be reduced (**Figure 3**). However, even after suitable techniques are developed, widespread use of CIDT as a replacement for culture-based techniques will still face significant challenges in food regulation and industry. International regulatory agencies with stakes in food safety such as Health Canada, US FDA, French agency for Food, Environmental and Occupational Health Safety (ANSES) are still in the initial phases of accepting WGS results as a replacement for more traditional techniques for outbreak investigations^2^. Some countries though, are much further along in this process than others (Allard et al., 2016). Using Canada and *Listeria* as an example, verification controls in processing environments is directly tied to consumer risk should the food be contaminated (Pagotto et al., 2011a). The ecology of *Listeria* in ready-to-eat products with respect to foods capable (or not) of supporting growth (or survival) may be elucidated through the use of WGS. Metagenomics has been used to help determine aspects related to enrichment (Ottesen et al., 2016); interference to even the ability of only detecting a single species when more than one may be present (Ottesen et al., 2016). A relatively recent study investigated the efficacy of NGS techniques to detect food pathogens (Leonard et al., 2015). An FDA culture-based protocol was performed on spinach spiked with STEC in addition to shotgun metagenomics sequencing. In particular, the study aimed to address limits of detection, sensitivity and specificity levels. The authors reported an expected level of contamination (approximately 10 cfu/100 g) and were able to accurately detect strain-level and virulence information within 8 h of enrichment at a sequencing depth of 10 million reads.

Next-generation sequencing-based approaches such as WGS rely on sequencing technologies, bioinformatics pipelines (Lambert et al., 2015) and high-quality reference databases (Allard et al., 2016). Therefore, widespread acceptance of WGS results, as a complete replacement for traditional molecular techniques must likely precede the introduction of shotgun metagenomics or targeted-amplicon approaches in food outbreak investigations and compliance testing. There are, however, many instances where metagenomics are being used in the food industry for rapid screening and research, but for regulatory testing purposes and for outbreak investigations, presumptive positive results must still be culturally confirmed^3^.

Metagenomics is currently being used in both the food industry and in research for many applications including: taxonomic profiling of microbiological food products and supplements, directing efforts to improve culture techniques, identification of non-culturable or fastidious pathogens and detection of co-contamination. Targeted-amplicon analyses for example, are well-suited to study, characterize and catalog changes in the bacterial populations that take place during fermentation reactions and long-term storage of traditional fermented foods such as soft cheese (Escobar-Zepeda et al., 2016), kimchi (Jung et al., 2011; Park et al., 2012), and kefir (Leite et al., 2012; Korsak et al., 2015). Targeted-amplicon analyses are also suited to evaluate the composition of probiotic supplements (Bergholz et al., 2014; Morovic et al., 2016). This technique, when used to evaluate probiotic supplements, could be of particular interest to regulatory bodies, since based on preliminary work, many of these products contain neither the number nor species of probiotic bacteria claimed on the label (Morovic et al., 2016).

Targeted-amplicon and shotgun metagenomics have also both been used to improve culture-based enrichment techniques by allowing for detailed characterization of population dynamics of the background microbiota during enrichment (Gorski, 2012; Ottesen et al., 2013, 2016; Jarvis et al., 2015; Zilelidou et al., 2016). When food items are added to a non-selective media, microbes from the background microbiota can co-enrich with pathogens interfering with detection and recovery of pathogens. For microbes from the microbial population closely related to the pathogen this can also be true of differential or selective media as well. As an example, tomatoes are well-known to be frequently implicated as the source of human *Salmonella* infections, however, isolating *Salmonella* from the tomato phyllosphere using a culture-dependent method has proven challenging (Ottesen et al., 2013). Shotgun metagenomics sequencing of enrichment media revealed that *Paenibacillus* spp. readily outcompetes and may even kill *Salmonella* during the enrichment phase suggesting that an alternate enrichment media be used when investigating tomato contamination (Ottesen et al., 2013). A similar enrichment problem occurs with cilantro, where traditional pre-enrichment steps encourage the growth of Gram-positive Firmicutes instead of Proteobacteria (*Salmonella*), suggesting that an alternate pre-enrichment media should be developed for cilantro testing (Jarvis et al., 2015). The usefulness of targeted-amplicon and shotgun metagenomics sequencing in informing enrichment strategies has similarly been demonstrated in *L. monocytogenes* (Ottesen et al., 2016) and *E. coli* (Margot et al., 2016).

The use of CIDTs are much more readily acceptable for outbreak delineation and regulatory testing for non-culturable or fastidious foodborne pathogens such as viruses (Vinjé, 2014) and parasites (Buss et al., 2013). For example, noroviruses, which until very recently were non-culturable (Ettayebi et al., 2016), are currently detected by Sanger sequencing of partial regions of the polymerase and capsid sequences, directly from clinical samples (Vinjé, 2014). However, the same techniques are rarely successful on food samples since the viral titer in the food is generally too low (Gentry-Shields and Jaykus, 2015; **Figure 3**). Sanger sequencing of norovirus requires several PCR rounds to obtain usable information, and still only yields partial genome sequences (Verhoef et al., 2012). To successfully delineate outbreaks with 100% specificity, a full capsid sequence is required (Nasheri et al., 2017). Currently, several WGS workflows exist for norovirus analysis, including the sequencing of several overlapping PCR fragments (Kundu et al., 2013; Won et al., 2013), target enrichment of the norovirus genome using custom designed RNA baits (Brown et al., 2016) and metatranscriptomics approaches (Bavelaar et al., 2015; Nasheri et al., 2017; **Figure 3**). The metatranscriptomics approach has the advantage of being rapid and further, a *de novo* assembly can be obtained. This approach is thorough and additionally, allows for the presence of other co-infections to be detected from the same sequence data (Nasheri et al., 2017). Metagenomics sequencing has also been used to investigate various food products for the presence of potential or emerging pathogens in the absence of a defined outbreak (Kawai et al., 2012; Aw et al., 2016). A shotgun metagenomics approach has recently identified several human and animal viruses in fresh produce (Aw et al., 2016), and it can be expected that this type of analysis will be repeated for other food products to provide greater insight into the scope of viral contamination of the food supply. Non-culturable parasites present similar challenges to non-culturable viruses; shotgun metagenomics has also provided a suitable solution for detecting parasitic contamination. Between 2008 and 2010, Japan experienced multiple outbreaks of gastroenteritis that included more than 1300 cases caused by an unknown etiological agent. Shotgun metagenomics was used to demonstrate that a parasite (*Kudoa septempunctata*) was the likely cause of the outbreak (Kawai et al., 2012).

The current regulatory dilemma is that it is impossible to tell directly from the presence of DNA if an organism is viable or not, and although DNA degrades over time, false positive rates of pathogen identification due to the detection of naked DNA have the potential to be quite high. This is where food differs from clinical samples. If pathogen DNA is detected in clinical samples, there is a very high-probability that the pathogen is alive and replicating in the patient. However, finding pathogen DNA in foods has quite a different interpretation. A well-known example is *Listeria* in smoked fish, where its DNA can be present due to dead cells but molecular methods alone would generate incorrect compliance activity based on a PCR-based method (Gambarin et al., 2012; Law et al., 2015). In addition, food-processing techniques (such as thermal, high-pressure, radiation exposure, and others) are known to kill bacteria, but leave detectable pathogen DNA in the food matrices. This problem with false-positive detection is a significant hurdle to overcome before metagenomics approaches will be useful in outbreak delineation or compliance testing in the food industry (Law et al., 2014). Moreover, when secondary analyses including virulence or AMR gene detection are applied to the metagenomics data, and particularly if the genes are known to be mobile, there is a current lack of bioinformatics pipelines available to accurately predict if the virulence or AMR genes belong to the pathogen, or rather to the background microbiota. It has been suggested that the alternative use of mRNA could contribute to solving this problem, although, its ability to persist in various samples has also been implied (Leimena et al., 2013).

### Diagnostic Case Studies Revealing the Usefulness of Metagenomics and Comparable Techniques

Numerous studies have investigated the capacity of diagnostic metagenomics for infectious diseases from clinical specimens; these studies have proven the methodology clinically useful from an analytical perspective and secondly, in an appropriate timeframe that can nevertheless yield positive patient outcomes (Nakamura et al., 2008; Wilson et al., 2014; Mongkolrattanothai et al., 2017). Both high-throughput targeted-amplicon and shotgun metagenomics sequencing have been performed on biological specimens for unknown pathogen detection either for research purposes or when all previous diagnostic tests have proven inconclusive. Importantly, the use of both high-throughput sequencing strategies to determine etiological causation is highly experimental due to inherent limitations (discussed in upcoming section) and typically only performed on select cases in diagnostic settings. Preceding widespread adoption and implementation of these methodologies, case studies are needed to evaluate concordance and compatibility with conventional diagnostic methods. Interpretation and reporting guidelines, in addition to criteria are likewise required prior to real-time use of this technology.

From a diagnostics perspective, metagenomics sequencing was first employed to detect human-associated viruses (rather than bacteria), which is unsurprising given the challenges associated with culturing most viruses. Throughout the last decade, viral metagenomics (viromics) has been used as a tool to diagnose cases of acute gastroenteritis and further, to determine etiological causation retrospectively in gastroenteritis outbreaks (Finkbeiner et al., 2009; Smits et al., 2014). This technique has proven particularly useful in determining causative agents whereby culture yielded negative results. It is hypothesized that novel viruses may account for a fraction of idiopathic gastroenteritis cases especially given that a large portion of global viral diversity has yet to be discovered (Anthony et al., 2013); hence methodologies incorporating “unbiased” and systematic high-throughput sequencing have the capacity for novel virus detection and discovery, which may have otherwise evaded routine diagnostic testing. In this regard, an early study aimed to characterize the viral populations present in pediatric diarrheal specimens (Finkbeiner et al., 2008). Interestingly, this study adapted a “micro-mass sequencing” approach. This included a minimal sample quantity (<100 mg stool), minimal sample purification and minimal sequencing (e.g., 384 reads per sample). Known enteric viruses including rotaviruses, caliciviruses, astroviruses, and adenoviruses were detected in addition to several sequences from at least nine putative novel viruses. Recently, a shotgun metagenomics approach was applied retrospectively to detect novel and known viruses associated with gastroenteritis outbreaks (Moore et al., 2015). Though the study failed to identify any novel pathogens, eight viruses, and one parasite were detected. The authors’ concluded that metagenomics could be useful to detect pathogens whereby routine testing has failed.

An early study tested the capability of shotgun metagenomics sequencing to detect bacterial pathogens during a case of acute gastroenteritis following culture based diagnostics where no candidate pathogens were identified (Nakamura et al., 2008). This case reported 156 *Campylobacter jejuni* sequences from a stool sample obtained when the patient was symptomatic versus no sequences identified in a sample obtained 3 months post-illness. This investigation represents one of the first proof-of-concept studies to be conducted in a clinical diagnostic setting.

A relatively recent case study emphasizes the clinical relevance of metagenomics as a diagnostic assay (Wilson et al., 2014). Briefly, a 14-year-old boy presented to a medical facility three times with complaints of fever and headache that ultimately advanced to hydrocephalus and status epilepticus. Though diagnostic workup was inconclusive on several occasions, an MRI eventually revealed encephalitis-like indications. CSF and serum specimens were subjected to shotgun metagenomics sequencing. Within 48 h of specimen receipt, bioinformatics analysis of CSF revealed a high abundance of sequences with homology to the *Leptospiraceae* family and mapped most closely to the pathogenic *Leptospira borgpetersenii* genome. The patient was therefore treated for neuroleptospirosis with intravenous penicillin G and markedly improved. In this context, treatment was initiated on the basis of metagenomics evidence and congruent clinical presentation prior to the completion of validated confirmatory testing. Five months post-treatment, targeted PCR and Sanger sequencing identified *L. santarosai* as the infectious agent. This case highlights various advantages and limitations associated with diagnostic metagenomics that will therefore be discussed in the following section.

Diagnosis of brucellosis historically has been a challenge as this illness presents with a continuum of clinical manifestations. In addition, diagnosis is further limited by current diagnostics via their inadequate sensitivity and specificity (Mongkolrattanothai et al., 2017). A recent report described a case whereby a shotgun metagenomics analysis of the patient’s CSF was used to provide an accurate diagnosis (via *Brucella* spp. detection) and directed towards appropriate antibiotic therapy ultimately leading to a favorable patient outcome (Mongkolrattanothai et al., 2017). This study led to the development of a validated diagnostic assay in the CLIA-certified University of California, San Francisco clinical microbiology laboratory^4^.

A recent study aimed to assess the concordance between various diagnostic (PCR, qPCR) and NGS techniques (16S rRNA targeted-amplicon and shotgun metagenomics) for *C. difficile* infection (Zhou et al., 2016). Intriguingly, of PCR and qPCR positive *C. difficile* samples, this microbe was detected in 90.9% of samples via 16S rRNA analysis versus 86.3% with shotgun metagenomics. Moreover, *C. difficile* was co-detected with several known enteric pathogens such as norovirus and sapovirus which adds further credence to the efficacy of NGS CIDTs to firstly, detect foodborne pathogens in an unbiased technique and secondly, to detect polymicrobial infections. This study reflects current limitations of metagenomics techniques in that their sensitivity is still relatively low.

We mentioned previously that high-throughput targeted-amplicon sequencing is at the moment largely research based. A recent study described the usefulness of 16S rRNA targeted-amplicon sequencing in an exploratory setting (Almonacid et al., 2016). This study utilized 46 bacteria and archaea encompassing 15 genera and 31 species of microbes in the human gastrointestinal tract selected on the basis of clinical relevance including pathogenic, commensal, and probiotic prokaryotes. A bioinformatics annotation pipeline specifically tailored to have high prediction performance was applied. For example, taxonomies were reported based on 100% identity over the entire 16S rRNA V4 region and curated databases were procured for each taxon using various optimizing parameters such as sensitivity, specificity, precision and a negative predictive value. Applying a threshold of 90% for each parameter, 28 of 46 targets could be detected. Microbiome specimens from a cohort of 897 healthy persons were used to define a reference range that could be used to establish clinically applicable relative abundances for each target. The authors concluded that their assay accurately identified and quantitated all targets and known pathogens from each sample (clinical or synthetic). Thus, assays such as this may facilitate improvements to patient diagnosis, treatment, monitoring, and epidemiological study.

The use of diagnostic metagenomics is less established in the framework of parasitic infections; however, studies are beginning to investigate their role (or merely, presence) in the human gastrointestinal tract, in addition to elucidating genomic epidemiology. Moore et al. (2015) also detected a parasitic candidate – *Dientamoeba fragilis*. While the role of *D. fragilis* in causing gastroenteritis remains unclear it is suggested that in the absence of other enteric pathogens that this parasite could be considered a causative agent (Barratt et al., 2011). This study also revealed an interesting phenomenon: while *D. fragilis* was detected in 10.9% of undiagnosed outbreak samples, a higher frequency was reported (44%) in pediatric samples. A more recent study applied several diagnostic techniques (metagenomics, microscopy, and multiplex PCR) to four diarrheal samples as a means to detect multiple pathogens simultaneously (Schneeberger et al., 2016). Metagenomics detected 8–11 plausible enteric pathogens in all samples. Specifically with bacterial pathogens, diagnostic agreement between PCR and metagenomics was high though metagenomics did identify several bacteria not detected by PCR. Perhaps more interesting, however, was the finding that microscopy could detect some helminth and protozoan infections that metagenomics could not, again reflecting sensitivity issues inherent of metagenomics techniques. Parasitic metagenomics has also shown effective in the investigation of *Cyclospora cayetanensis* – a coccidian parasite responsible for several food and waterborne outbreaks worldwide (Cinar et al., 2015) and in a case study of Malayan filariasis caused by *Brugia malayi* (Gao et al., 2016).

### Technical Challenges of Metagenomics Sequencing and Analysis

The diagnostic applicability of clinical metagenomics presents with its own shortcomings and hence is currently limiting the widespread use of this approach in clinical and food microbiology laboratories. In this regard, the aforementioned neuroleptospirosis case highlights many key points with respect to the utility of metagenomics in a diagnostic setting. First, Wilson et al. (2014) reported that a diagnosis would not have been possible via conventional assays. As is the case with many infections, differential diagnoses can be extensive; consequently, cultures, serology, and pathogen-specific PCR can be difficult in determining etiological causation in cases of this magnitude. These difficulties would particularly benefit from the use of unbiased diagnostic methods. Second, reference databases (e.g., NCBI, UniProt for protein and SILVA for ribosomal RNA) are a cornerstone of NGS-based diagnostics, however, the level of curation within these databases is variable. In the absence of adequately curated databases, accurate diagnoses will not be possible and could be detrimental to patient outcome should a misdiagnosis occur. In the neuroleptospirosis case, shotgun metagenomics and bioinformatics analyses utilizing the NCBI reference database identified *L. borgpetersenii*; confirmatory testing revealed the causative agent to be *L. santarosai*. In this context, query sequence homology to a reference sequence should not be misconstrued as causation. Third, the exceptionally short TAT led to appropriate therapeutic management and thus clinically actionable results, which corresponds to our next discussion point – perhaps most importantly is the developmental need for analysis tools and bioinformatics pipelines modernized for the requirements of diagnostic laboratories. For example, tools for pathogen detection must provide analytical results while concurrently minimizing analysis time to allow for reasonable diagnostic TAT. Though several analyses software or platforms for microbial identification are available (Segata et al., 2012; Naccache et al., 2014; Wood and Salzberg, 2014; Flygare et al., 2016), appropriate use typically requires bioinformatics or computational biology expertise often not available in diagnostic laboratories. One such computational analysis pipeline recently developed, SURPI (Naccache et al., 2014), is designed for unbiased pathogen detection from shotgun metagenomics sequencing data of clinical specimens applicable to infectious disease diagnostics, public health surveillance and outbreak analysis. Currently, practical utilization of this technology is hindered by several computational and technical challenges of analyzing data accurately and in a clinically actionable timeframe. Specific requirements include access to high performance computing, as well as laboratory technicians that are cross-trained in bioinformatics (ability to generate data) and in biology (provide interpretation of the data). There is an important need to develop clinically useful pipelines such that metagenomics can be implemented as a diagnostic tool.

Not only are challenges associated with the analytical performance, but sample preparation and sequencing methodologies must also be standardized. Due to the infancy of this field, standardizations are still in progress and various ring trials and proficiency tests are underway to standardize various aspects pertaining to studies of this nature. For further information we direct readers to the following articles (Lesho et al., 2016; Mellmann et al., 2017). As outlined in **Tables 1, 2**, the choice of sequencing technique applied is largely dependent on the research or diagnostic goals while also considering advantages or disadvantages of each method. Other factors that can affect pathogen identification include as previously discussed, pre-enrichment steps, quality of DNA extraction, library preparation and chemistry. Furthermore, we are faced with questions pertaining to how to work with contaminants or host DNA, low biomass specimens, defining ideal read depths for particular biological specimens or food products and lastly, defining sequence number thresholds to confidently assign pathogenic etiology.

The presence of contaminants or host DNA poses serious challenges to metagenomics data analysis whereby in many instances, true (e.g., microbial) metagenomics data is overwhelmed by the abundance of host DNA. Considerations should be given to samples where eukaryotic cell content is expected to be high (e.g., biopsy). Similarly, variable host DNA content will be found in samples such as stool dependent on the consistency and quality (e.g., healthy, watery, or bloody). Studies have attempted to address the issue of contaminants (Salter et al., 2014) and host DNA (Hasan et al., 2016) with variable success. The primary concern, however, are methodologies surrounding removal of these products and their plausible effect on microbial DNA abundance or quality in a given sample.

While we frequently refer to metagenomics as an unbiased methodology, there are biases that need to be considered to ensure that all microbes of interest are captured adequately in the sequencing output. As an example, interrogating the DNA or RNA virome using a metagenomics approach (e.g., viromics) requires complex wet-laboratory procedures that are considerably distinct from that of a sample workup for a general metagenomics assay of ‘all’ microbes. Viruses have significantly smaller genomes as compared to prokaryotes or eukaryotes, thus their genomic content represents a microscopic fraction of total DNA or RNA in a given sample (Kleiner et al., 2015). Moreover, characteristics of viral particles are considerably diverse (e.g., size, density, structure, and others) and also include phages. In this context, an intricate series of sample and nucleic acid treatments must be conducted to ensure viral particles are amply interrogated with high sensitivity in a metagenomics assay (Thurber et al., 2009).

Within a given specimen, a broad range of taxon abundances are observed. Though shotgun metagenomics can produce near-complete whole-genome coverage of highly abundant microbes, etiological agents are not always among the most abundant. For example, while the infective dose (in colony forming units) is 10^7^–10^9^ for enterotoxigenic *E. coli, L. monocytogenes* is 10^3^ and *Salmonella* spp. is only 4–50 (Todd et al., 2008). Achieving sufficient sensitivity in shotgun metagenomics studies has proven to be a challenge, particularly with respect to low abundance microbes and gene level identification. For example, data mining for AMR, toxin or virulence genes has proven difficult. This was well-established in a retrospective metagenomics study that aimed to investigate an outbreak of STEC O104:H4 (Loman et al., 2013). The authors’ reported that the outbreak strain was recovered to near completeness (full genome breadth) from 10 samples (of 45) at >10-fold coverage and from 26 samples at >1-fold coverage. The shiga-toxin gene, however, which was identified in 100% of samples via culture-based methods was only detected in 27 of 40 (67%) STEC-positive samples. This study highlights some of the challenges of metagenomics related to gene level sensitivity. Further examination, particularly into the acceptable sequencing depth to ensure high sensitivity and specificity is warranted.

Metagenomics has already proven an effective complement to conventional diagnostics in complex cases and outbreaks (Loman et al., 2013; Ruppé et al., 2016; Langelier et al., 2017) though much remains to be elucidated prior to the widespread adoption of metagenomics in diagnostic and public health laboratories. Therefore, while high-throughput sequencing of biological specimens has a promising future, its utility is unlikely to become a standard and approved CIDT method in the imminent future.

## Influence of Culture-Independent Diagnostic Testing on Human Disease Surveillance

### Pathogen Surveillance and Subtyping

Metagenomics sequencing of a clinical or environmental sample could offer a universal test for pathogen detection, clinical diagnosis, as well as a subtyping test for routine surveillance activities. Surveillance for foodborne infections historically has required the culturing of an isolate in order to perform the appropriate genotypic or phenotypic characterization, yielding a phenotype or genetic fingerprint capable of making detecting and resolving outbreaks possible. By tracking foodborne pathogens along the farm to fork to clinical specimen continuum, it is possible to monitor trends over time, track which foods are capable or implicated in causing illnesses and detecting outbreaks.

Similar to trends occurring in infectious disease diagnostics, techniques for subtyping have enhanced the ability to distinguish between epidemiologically linked isolates from identical microbial species hence improving outbreak detection, surveillance and the overall understanding of microbial epidemiology. Molecular subtyping techniques in routine use include PFGE and MLVA; PulseNet Canada^5^ (national real-time surveillance and outbreak response) has relied on these for nearly two decades but is presently transitioning to the use of WGS. The other national surveillance systems dedicated to foodborne disease in Canada, the National Enteric Surveillance Program^6^ (weekly trend analysis at the species or serotype level of bacterial, viral and parasitic enteric pathogens) and FoodNet Canada^7^ (sentinel site-based surveillance system measuring burden of illness, attribution studies and food safety policy recommendations) are also transitioning from molecular subtyping to WGS.

### Genomic Epidemiology

Recent improvements of sequencing technologies and streamlined bioinformatics tools have not only advanced clinical diagnostics but are also transforming public health. WGS is increasingly implemented in epidemiological study, outbreak detection and surveillance of foodborne bacteria. Genomic epidemiology refers to the use of WGS to investigate epidemiological features. The listeriosis outbreak described above was the first application of WGS in during an active foodborne outbreak investigation (Gilmour et al., 2010). This study was seminal in bridging the gap between WGS and public health in real-time. Further, the initial study to utilize WGS for source attribution was performed during the 2009–2010 *S. enterica* serotype Montevideo outbreak (Lienau et al., 2011). The source was traced to red and black pepper that was used in the production of Italian-style spiced meats in a New England processing facility. WGS has since been executed in manifold outbreak and surveillance analyses.

Between 2010 and 2015, numerous severe illnesses associated with a complex multi-state listeriosis outbreak were reported and linked to two facilities of a large commercial ice cream producer as the source of *L. monocytogenes* (Jackson et al., 2016). This outbreak is highlighted here particularly due to the unusual length of the outbreak. Specifically, guidelines pertaining to listeriosis outbreak investigations have generally used a 120-day window (versus 16-days for other foodborne pathogens) for inclusion of suspected cases attributed to its psychrotrophic nature and viability in cured and processed food products with longer shelf lives. WGS routine implementation for all clinical, food and food processing environmental isolates, for current and retrospective cases led to strong evidence supporting a lengthy listeriosis outbreak. Traditional investigation guidelines and culture-based subtyping methods (e.g., PFGE) would not have been able to unequivocally link *Listeria* isolates to an outbreak cluster. The high discriminatory power of WGS combined with strong epidemiological evidence will inevitably lead to a higher proportion of detected and resolved outbreaks and a concomitant lower number of patients within each cluster, thus allowing for contaminated products to be removed from commerce more promptly.

### Metagenomics is Capable of Providing Informative Subtyping Data

Advancements have clearly been made with respect to pathogen detection via the application of molecular diagnostic techniques such as PCR. These advancements are driven by the capacity to bypass the need for pathogen culture and isolation. Current surveillance methodologies (e.g., PFGE, MLVA, MLST, and WGS) however, are reliant upon the presence of isolates. Techniques used in public health surveillance for disease tracking and subtyping therefore necessitate adaptation to the culture-independent trend.

Rapid infectious disease diagnostics would particularly benefit from the ability to directly subtype pathogens from complex clinical specimens. While subtyping methods such as targeted-amplicon sequencing, FISH (Splettstoesser et al., 2010) and repetitive element sequence-based PCR (Hahm et al., 2003) may be able to interrogate and subtype microbes, their ability to do so at an adequate taxonomic resolution renders these assays less efficient in differentiating between subtypes of a specific species, compared to gold standard methods such as PFGE. Metagenomics sequencing of clinical specimens represents a plausible epidemiological and subtyping tool. Hundreds to thousands of sequence reads for a particular species are generated through metagenomics sequencing thus potentially providing sufficient informative data for subtyping. Genome coverage of a given microbe, however, is difficult to predict and often the pathogen may not be the most abundant microbe in a given specimen due to the pathogen’s infective dose. Also, some serogroups are unable to be typed, for example “O rough” STEC (Chattaway et al., 2016). In recent years, the widespread usage of research-based metagenomics has coincided with the development and application of a plethora of novel analysis techniques, some of which are well-suited to type (e.g., interrogate the microbe below species level taxonomic resolution) microbial strains (Zagordi et al., 2011; Hong et al., 2014; Ahn et al., 2015; Cleary et al., 2015; Sahl et al., 2015; Joseph et al., 2016). Numerous analysis software have been developed and similarly been shown to achieve adequate sensitivity and specificity for pathogen identification. As of yet, metagenomics to our knowledge has not been utilized in a communicable disease tracking perspective.

### Changing Trends in Public Health Surveillance

Effective detection of outbreaks, particularly broadly disseminated outbreaks caused by the commercial distribution of contaminated foods is largely dependent on subtyping isolates from a sizeable proportion of cases. As diagnostic laboratories are in the process of shifting from diagnostic tests (culture-based or WGS) yielding isolates to CIDTs (molecular or serological), it is essential to maintain a system whereby subtyping can still be performed on a large percentage of positive specimens.

With changing diagnostic practices, several options to maintain the capability of foodborne surveillance, outbreak detection and source attribution are possible. First, clinical (and food) microbiology laboratories could perform reflex culturing of biological specimens (or food) that test positive via CIDTs such that positive isolates can still be submitted to public health laboratories for subtyping or other culture-based tests such as WGS (Cronquist et al., 2012). Second, clinical laboratories could alternatively submit biological specimens to public health laboratories that have tested positive using CIDTs. Lastly, culture-independent subtyping and streamlined bioinformatics analyses could be developed for both public health and clinical microbiology laboratories; this would be particularly beneficial for specimens incompatible or not optimal with culture (e.g., fecal swabs; Kotton et al., 2006). Hence, while any of these methods would require substantial restructuring of national and international surveillance infrastructure it would in theory overcome the prospective predicament whereby public health laboratories would not have access to a considerable portion of isolates and thus capacity for precise outbreak detection and source attribution. If, however, culture were rendered obsolete prior to the implementation of any of the above-listed scenarios, it would inevitably be detrimental for public health surveillance. Additionally, the absence of isolates (and a biobank of historical isolates) will make retrospective studies of outbreaks challenging. In this regard, regulatory groups have been created in both Canada and the US to address the concern pertaining to a lack of enteric isolates available for further characterizations at public health laboratories. The US has created an interim recommendations document^8^ to ensure that isolation is attempted or that positive CIDT specimens are retained – Canada is in the process of developing analogous guidelines.

## Transformation of Antimicrobial Susceptibility Testing and Influence on Antimicrobial Resistance Surveillance Systems

A limited number of conventional growth-based methods for AST have persisted in routine usage throughout the transformation of diagnostic microbiology. Included among these are disk diffusion (e.g., Kirby-Bauer) strategies and broth microdilutions. The latter of which has achieved gold standard status; thus, novel AST methods are compared to the efficacy of broth microdilutions from development through clinical trial. At present, AST is either accomplished via the above-listed conventional manual methods or growth-dependent automated AST systems including the Vitek System (bioMerieux, France), Avantage Test System (Abbott Laboratories, Irving, TX, United States), Phoenix (BD Biosciences, Cockeysville, MD, United States) and others, all of which are based on broth microdilution testing (Van Belkum and Dunne, 2013). More recent growth-based AST techniques have also been developed that generally employ innovative methods; these include MALDI-TOF mass spectrometry, microfluidics (NanoDrop BMD), isothermal microcalorimetry, real-time microscopy, and others.

### Whole Genome Sequencing Antimicrobial Resistance Gene Detection

With the rising usage of molecular CIDTs and NGS strategies in clinical diagnostics, speculation exists regarding the potential feasibility of NGS methods or other advanced technologies, as aforementioned, replacing growth-based AST. Assessments have been completed for some species [e.g., *Salmonella* (McDermott et al., 2016) and *Gonorrhea* (Demczuk et al., 2015)] and it has been suggested that resistance genes can be accurately detected and further, the presence of genes and mutations associated with AMR has been shown to have a high correlation with phenotypic antimicrobial susceptibility profiles. As an example, WGS was recently applied to non-typhoidal *Salmonella* isolates (McDermott et al., 2016). The authors reported an overall 99% concordance rate between genotype and phenotype; for most classes of antibiotics, concordance was closer to 100% while lower for aminoglycosides and beta-lactams. Though inferring AST via WGS is becoming more common in diagnostic laboratories, to date, few studies have performed large-scale sequencing projects to investigate the utility of WGS to complement or replace conventional AST in routine laboratory workflows. Nonetheless, limitations concerning particular phenotypic-only traits that can’t be determined exclusively from WGS or other NGS methodologies will need to be addressed.

### Metagenomics Antimicrobial Resistance Gene Detection

As discussed previously, conventional approaches used to determine AMR are generally reliant upon growth-based tests and further, are heavily targeted to testing human pathogens. The methodological spectrum for AMR gene detection must be expanded to overcome imperative limitations: relatively few bacterial microbes can be cultured (Eckburg et al., 2005) and commensals are thought to comprise a resistance gene pool (resistome) that can be transferred to pathogens (Davies and Davies, 2010). Hence, metagenomics has the capacity to overcome challenges associated with traditional AST. At present, both shotgun metagenomics sequencing and functional metagenomics have been utilized to interrogate the resistome.

The use of shotgun metagenomics sequencing to identify AMR genes is promising and studies are now beginning to elucidate their plausible role in surveillance. In this technique, metagenomics reads are mapped against a database containing a comprehensive catalog of known AMR genes; notable examples include CARD (Jia et al., 2017), ARDB (Liu and Pop, 2009), Resfams (Gibson et al., 2014), and MEGARes (Lakin et al., 2017). Alternatively, metagenomics reads can be assembled into contigs and next compared to a functional annotation database. A recent study surveyed the metagenomes of several ecological niches including the human gastrointestinal tract, water, animals and others for AMR genes (Fitzpatrick et al., 2016). A large abundance of AMR genes were detected in the human gastrointestinal tract and variably identified in other metagenomes. Nonetheless, the authors emphasized the importance of difficulties associated with shotgun metagenomics detection of AMR genes. First, similar to determining microbial abundance reference ranges (from a diagnostics perspective), limits of detection need to be established in order to have sufficient coverage with the capacity to detect rare AMR genes or in complex metagenomes. Second, methods to normalize data to overcome variable microbial diversity and genome sizes will also be required. Third, the biological features inherent of AMR genes (e.g., often carried within the mobilome or other transmissible genetic elements) render sequencing and data analysis difficult due to their repetitive nature.

Numerous studies have applied shotgun metagenomics sequencing to detect AMR genes. The *C. difficile* study discussed above identified a total of 27 AMR genes and 55.6% of samples contained a minimum of 1 gene (Zhou et al., 2016). The most dominant AMR genes encoded cephalosporin (*Bl2e_cfxa*; 25.9%) and tetracycline (*tetQ*; 25.9%) resistance whereas macrolide (*ermA, ermB, ermF, ermG*) resistance was variable ranging from 3.7 to 11.1% of samples. It was also shown that WGS of isolates predicted AMR phenotypes with high accuracy. A recent study explored the utility of shotgun metagenomics to detect MDR pediatric bacterial infections, specifically, methicillin-resistant *Staphylococcus aureus*, vancomycin-resistant *Enterococcus* and MDR Enterobacteriaceae (Andersen et al., 2016). The study included three cohorts – high-risk inpatients, low-risk outpatients and controls. Though the potential for MDR bacteria was increased in inpatients and outpatients compared to controls, no differences were detected between inpatients and outpatients. A noteworthy observation was that 53% of inpatients were colonized with an MDR bacterium that culture failed to identify.

Functional metagenomics on the other hand is an exceedingly powerful technique attributed to its capacity to discover novel and highly divergent AMR genes (Perry and Wright, 2014). This method involves cloning total community genomic DNA into an expression vector and transformation into a susceptible expression host (e.g., *E. coli*). The transformant library is then assayed for AMR by culturing on selective media – persistent AMR genes are sequenced and annotated. Therefore, this method is advantageous, ascribed to the possibility to determine genotypic and phenotypic traits.

As NGS-based methodologies like WGS are proving useful in clinical and public health laboratories, we expect that other advanced sequencing techniques will similarly be effective in amalgamating molecular AST and phenotypic susceptibilities thereby allowing for a complete bypass of microbial culture. In this regard, NGS supplemented with metatranscriptomics or proteomics can be more informative as it allows for description of both gene presence and expression (Cohen et al., 2015; Perez-Llarena and Bou, 2016). A combinatorial ‘omics’ approach incorporating any of the above-mentioned assays may be initially computationally laborious due to a paucity of streamlined analysis techniques and standardization. However, upon analytical validation, such an approach may still be less laborious than conventional culture-dependent testing.

### Current Condition of Antimicrobial Resistance Surveillance

Public health laboratories routinely track specific characteristics of bacterial pathogens that are implicated in infection, thus effectively allowing for increased understanding of bacterial pathogens and their epidemiology. Among these characteristics include monitoring AMR of common enteric pathogens such as *Salmonella* spp. and *Campylobacter* spp., as well as virulence profiles in for example STEC. At the Canadian public health level, AMR surveillance is conducted through the Canadian Integrated Program for Antimicrobial Resistance Surveillance (CIPARS)^9^ in combination with FoodNet. Importantly, though AMR in a clinical setting is largely associated with the utilization of antimicrobials for the treatment of infections, their use in agri-food production is also known to contribute to the resistant microbe pool. Resistant bacteria are associated with more severe disease (van Duin and Paterson, 2016) and poor patient outcome, hence monitoring for AMR is critical. Accordingly, AMR or MDR bacteria have caused several recent foodborne outbreaks (Cartwright et al., 2016; Gieraltowski et al., 2016; Kawakami et al., 2016).

Irrespective of how and when NGS technologies will be capable of rapidly and accurately detecting AMR and associated susceptibilities, difficulties in public health AMR surveillance will be apparent. With a growing number of clinical and food microbiology laboratories opting for utilization of CIDTs for diagnostic purposes, laboratories are bypassing the need to culture microbes; this may affect public health surveillance of AMR trends which continue to use isolates as the foundation for AMR surveillance programs and assessment of phenotypic resistance to antimicrobials. CIDT for enteric pathogens that yield no isolate and potentially no specimen for public health laboratories does pose an important challenge, however, actions can be taken to maintain adequate AMR surveillance. Gonorrhea, for example, represents a proof-of-principle for overcoming a lack of isolates in the context of surveillance. In particular, routine testing of gonorrhea has been performed by NAATs for some time due to the higher sensitivity and specificity. Guidelines have been documented regarding sentinel surveillance mechanisms to continue monitoring AMR trends^10^. Moreover, CIDT techniques could also be developed that have the capacity to test for known AMR though culture will still be systematically needed to detect novel and emerging resistance mechanisms.

## Future Challenges of Metagenomics in Diagnostic and Public Health Settings

Whole genome sequencing as a complement to conventional culture-based, molecular or serological CIDTs in real-time serves as an empirical model for the use of future techniques (Jackson et al., 2016). From lessons learnt via WGS application, metagenomics and other NGS techniques should move into front-line laboratories in the near future. Initial proof-of-concept studies (Gilmour et al., 2010), retrospective analyses (Loman et al., 2013), validation processes and real-time implementation of WGS (Jackson et al., 2016) in public health surveillance and outbreak response will undoubtedly help enhance acceptance of the aforementioned metagenomics methodologies from the medical and public health community at large. Standardization is an essential element for implementation of any method set to become the next “gold standard.” Various WGS studies have been undertaken of historical clusters and sporadic cases to determine concordance of WGS data with traditional subtyping and epidemiological data; in many instances, WGS provided higher discriminatory power than traditional subtyping methods and thus resulted in many occurrences whereby isolates were either included or excluded from an outbreak due to WGS and supportive epidemiological data, if present. To date, isolate cluster inclusion/exclusion criteria remains a moving target due to sizeable differences in biology amongst foodborne pathogens. Although WGS is part of the public health toolbox, it remains to be standardized in such a way that it can be applied to all foodborne pathogens. As a result, regular cluster audits to assess analysis pipelines for performance in addition to adequacy of quality control and assurance metrics for sequencing and all analysis outputs should be routinely conducted. Furthermore, the transition to add WGS as a public health epidemiology tool has been an internationally driven effort with organizations and partners from every field – primary health care, academia, industry and all government tiers. For wide adoption of new methods, involvement of the international community is key. Each of these aforementioned guidelines will be paramount to ensure cluster definitions remain robust in the context of genomic epidemiology. Another crucial aspect of implementing new technologies is knowledge translation. Currently, large efforts are being directed at educating and communicating the use of WGS in public health surveillance and outbreak response. Not only will laboratory technicians be required to advance their skillsets, but also policymakers and media will require training on how to interpret highly technical data and communicate it to the public (Jackson et al., 2016). As we enter this new era of “omics” in public health, the aforementioned criteria will be instrumental in guiding and implementing unbiased NGS-based CIDT methods in foodborne disease surveillance and outbreak response.

As mentioned previously, preceding widespread implementation of metagenomics sequencing and analysis in diagnostic and public health settings, bioinformatics encompassing algorithms, software and pipelines will need to be developed, validated and standardized through various ring trials and retrospective analyses. Perhaps the largest obstacle faced by the field of metagenomics is the lack of universal analyses or pipeline recognized as the status quo. In this regard, we previously discussed the significance of curated sequence databases to appropriate patient diagnosis. The GMI^11^ consortium has begun to address this issue via the creation of a global database that house uniquely identifiable microbial genomic data in combination with high quality meta- and epidemiological data. This drive will assist in the global amalgamation of microbial WGS and metadata in an easily accessible portal ultimately enhancing global surveillance of infectious microbes and emergent pathogens. Moreover, such a global database will similarly be influential in data mining investigations as they relate to gene level comparisons such as AMR, virulence, and environmental fitness.

Procurement of regulatory approval for routine implementation of NGS-based technologies in diagnostic, public health and food safety laboratories will be onerous owing to various difficulties inherent of such an assay. First, metagenomics dataset contain identifiable information. As discussed previously, many biological specimen types have high amounts of host DNA; thus, a large proportion of generated sequences will be of eukaryotic origin. In research– and diagnostic-based cases, host DNA is filtered from the dataset, however, issues are apparent with how best to protect patient privacy. There may be plausibility that genetically informative host sequence data could be used to screen against a panel of known disease-causing genetic variants. Providing patients with information pertaining to a potential genetic disease via such an assay is an ethical concern (Chrystoja and Diamandis, 2014). On the other hand, providing patients with their metagenomes may lead to precarious implications should patients seek to self-diagnose. Second, access to patent DNA from the human genome, food products and ingredients sequence data could pose unwarranted legal implications. The recovery of patented DNA sequences from food sources or ingredients such as genetically modified foods (crops or animals) may give rise to concerns of patent infringement (Chrystoja and Diamandis, 2014). Metagenomics may also be used for the screening detection of unauthorized GMO use and similarly aid regulation of the food industry, particularly with high import/export rate of global food products and ingredients. Additionally, metagenomics may allow for the detection of species fraud, food product mislabelling or incorrect claims (Corrado, 2016) that may be subject to increased scrutiny. Third, the detection of AMR or virulence genes may lead to undesirable outcomes. Specifically, rising concerns with AMR has led to the complete ban of antimicrobial sub-therapeutic use in food animals in the European Union The potential to identify AMR in food products may inhibit producers and commercial food processers from agreeing to regulations or policies related to metagenomics testing of food products in fear of repercussions in food trade and export. Overall, should metagenomics become a validated assay in clinical, food and public health settings its impact may be far-reaching in the realm of ethical and legal implications.

## Conclusion

The prospective use of diagnostic metagenomics and comparable techniques offers an assumption-free workflow (though biases are present with each methodology; **Table 1**) thus creating the ability to detect any and all pathogens (bacteria, virus, parasite, and others) from various biological specimens or food products. Through forthcoming improvements related to required technical expertise, throughput and cost-effectiveness of sequencing combined with enhanced and streamlined laboratory and bioinformatics pipelines, metagenomics will likely have a predominant role in the diagnostic and public health laboratory. We expect an automated metagenomics pipeline will complement and may even replace several methods currently employed in the diagnostic laboratory while concurrently providing additional information such as AMR, virulence, and genomic epidemiology. It is apparent that the functionality of diagnostic metagenomics has been established in research settings and further, in detecting etiological culprits of unidentified illnesses and outbreaks. We anticipate that within the next decade, detection and characterization of pathogens via metagenomics-based workflows will be implemented in routine usage in diagnostic and public health laboratories.

## Author Contributions

NK and AR developed the concept for the manuscript. JF, NK, JR, FP, and AR wrote the manuscript. All authors read and approved the final version of the manuscript.

## Conflict of Interest Statement

The authors declare that the research was conducted in the absence of any commercial or financial relationships that could be construed as a potential conflict of interest.

## Footnotes

1 http://www.hc-sc.gc.ca/fn-an/legislation/pol/policy_listeria_monocytogenes_2011-eng.php

2 http://www.fao.org/documents/card/en/c/61e44b34-b328-4239-b59c-a9e926e327b4/

3 http://www.hc-sc.gc.ca/fn-an/res-rech/analy-meth/microbio/index-eng.php

4 https://www.ciapm.org/project/precision-diagnosis-acute-infectious-diseases

5 https://www.nml-lnm.gc.ca/Pulsenet/index-eng.htm

6 https://www.nml-lnm.gc.ca/NESP-PNSME/index-eng.htm

7 http://www.phac-aspc.gc.ca/foodnetcanada/index-eng.php

8 https://www.aphl.org/aboutAPHL/publications/Documents/FS-Enteric_Pathogens_Guidelines_0216.pdf

9 http://www.phac-aspc.gc.ca/cipars-picra/index-eng.php

10 http://www.phac-aspc.gc.ca/std-mts/sti-its/cgsti-ldcits/

11 https://www.globalmicrobialidentifier.org

## Abbreviations

AMR: antimicrobial resistance
AST: antimicrobial susceptibility testing
CIDT: culture independent diagnostic test
CSF: cerebrospinal fluid
DNA: deoxyribonucleic acid
ELISA: enzyme linked immunosorbent assay
FDA: food and drug administration
FISH: fluorescent *in situ* hybridization
GMI: global microbial identifier
ITS: internal transcribed spacer
LAMP: loop mediated isothermal amplification
LFA: lateral flow assay
LLS: listeriolysin S
MALDI-TOF: matrix assisted laser desorption/ionization time of flight
MDR: multidrug-resistant
MLST: multilocus sequencing typing
MLVA: multilocus variable number tandem repeat analysis
NAAT: nucleic acid amplification test
NASBA: nucleic acid sequence based amplification
NGS: next-generation sequencing
PCR: polymerase chain reaction
PFGE: pulsed field gel electrophoresis
rRNA: ribosomal ribonucleic acid
SNV: single nucleotide variant
STEC: shiga-toxin producing *Escherichia coli;*
SURPI: sequence-based ultra-rapid pathogen identification
TAT: turn-around-time
WGS: whole genome sequencing
